# The Acid–Base Balance and Gender in Inflammation: A Mini-Review

**DOI:** 10.3389/fimmu.2018.00475

**Published:** 2018-03-07

**Authors:** Georges Jacques Casimir, Nicolas Lefèvre, Francis Corazza, Jean Duchateau, Mustapha Chamekh

**Affiliations:** ^1^Laboratoire académique de Pédiatrie, Hôpital Universitaire des Enfants Reine Fabiola, Université Libre de Bruxelles, Brussels, Belgium; ^2^Laboratoire de Médecine translationnelle, Centre Hospitalier Universitaire Brugmann, Université Libre de Bruxelles, Brussels, Belgium

**Keywords:** inflammation, homeostatic balance, neutrophils, monocytes, endothelial cells, gender differences, acid–base balance, mechanisms of inflammatory cascade

## Abstract

In humans, acid–base balance is crucial to cell homeostasis. Acidosis is observed in numerous inflammatory processes, primarily acute conditions such as sepsis, trauma, or acute respiratory distress where females tend to exhibit better prognosis compared with males. The mechanisms underlying these gender-dependent differences are multiple, probably involving hormonal and genetic factors, particularly the X chromosome. Although pH influences multiple immunological functions, gender differences in acid–base balance have been poorly investigated. In this review, we provide an update on gender differences in human susceptibility to inflammatory diseases. We additionally discuss the potential impact of acid–base balance on the gender bias of the inflammatory response in view of our recent observation that girls present higher neutrophilic inflammation and lower pH with a trend toward better prognosis in severe sepsis. We also highlight the potent role played by endothelial cells in gender differences of inflammation through activation of proton-sensing G protein-coupled receptors.

## Introduction

In both humans and animals, physiology and metabolism need homeostatic mechanisms (1) to maintain the stability of not only the intracellular but also the extracellular milieu and plasma, thereby guaranteeing long-term survival of multicellular organisms. Various factors including basic metabolism, diet, physical activity, and environmental aggressions are daily life triggers of many physiological imbalances. Inflammation is the main active response designed to avoid dramatic stress challenges to homeostasis in the setting of infections, tissue injuries, cancers, or large burns. To maintain a permanent cell homeostasis, different receptor types are capable of sensing short and significant fluctuations in certain variables, along with multiple messengers that likely modify cell recruitment (especially neutrophils from vessels and bone marrow), diapedesis of monocytes and macrophages, vascular permeability, and production of protein mediators like cytokines and chemokines. Neutrophils, the major inflammatory effector cells at the site of acute injury (abscess, surgical trauma, pleural effusion, etc.), are capable to live in extremely challenging conditions such as severe acidosis or lack of oxygen. Chronic inflammation and diseases with a similar response profile can be considered as a dysregulation of the defense mechanisms, causing deleterious tissue damage and increased morbidity and mortality. In these cases, different parameters may be chronically modified by inflammation, thereby exceeding normal ranges, without any possibility of returning to normal patterns when the system remains “locked.”

Mechanisms that control homeostasis could differ between males and females.

## Role of Acid–Base Imbalance as Trigger of Inflammatory Responses

During acute inflammatory processes, particularly infections and even more dramatically sepsis, the acid–base balance is usually severely challenged. The extracellular milieu’s pH interferes with a wide range of immunological functions (2, 3). The role of acid as trigger of cytokine production was already described in 1997. *In vitro* studies have emphasized, among others, the following associations: increased inflammatory cytokines, such as interleukin (IL)-1β, IL-6, or tumor necrosis factor-α (TNF-α), produced by mononuclear cells; neutrophil activation with upregulation of cluster of differentiation (CD)18 expression and hydrogen peroxide production; and maturation of human dendritic cells. More recently, in critically ill patients, a positive correlation was found between a strong anion gap and the concentrations of IL-6, IL-8, IL-10, and TNF-α (4, 5).

In the initial response to acute infection, the primarily phagocyte-based innate immune system likely plays a crucial role in managing the inflammatory process. A critical modulation of the early inflammatory process by metabolic acidosis may impact the prognosis of the sepsis. In a blunt trauma setting, upon admission, patients exhibit a significant base deficit that is associated with differential immune/inflammatory pathways, which may subsequently predispose patients to a more complicated clinical course (6). It could be postulated that the proton concentration that must be drastically controlled in life could represent a unifying signal inducing inflammation. Proton-sensing G protein-coupled receptors, including OGR1, GPR4, and TDAG8, were reported to prove highly significant for physiological pH homeostasis and inflammation control (7). Patients suffering from inflammatory bowel disease have been shown to express higher levels of these proton-sensing receptors in the mucosa compared to controls. It is interesting to note that proton pump inhibitors, which block gastric acid secretion, were shown *in vitro* to selectively inhibit TNF-α and IL-1β secretion by TLR-receptor-activated human monocytes, without any cellular toxic effects. They are thus considered as promising agents targeting severe inflammation (8), but might also account for increased susceptibility to infections in these patients (9). Proton pump inhibitors can also enhance the risk of *Clostridium difficile* infections (10).

GPR4, a proton-sensing receptor expressed in endothelial cells and other cell types, is fully activated by acidic extracellular pH. However, this product exhibits less activity at the physiological pH 7.4 and only minimal activity at a more alkaline pH (11). When varying GPR4 expression in human umbilical vein endothelial cells, it proves possible to induce a substantially increased expression of numerous inflammatory genes, such as chemokines, cytokines, adhesion molecules, nuclear factor kappa B (NF-κB) pathway genes, prostaglandin-endoperoxide synthase 2, and stress response genes. This also applies to human lung microvascular endothelial cells and pulmonary artery endothelial cells. While acidosis-induced GPR4 activation stimulates the expression of numerous inflammatory genes in endothelial cells, it has been possible to suppress this inflammatory response by small molecule inhibitors of GPR4, which suggest a potential therapeutic value of such agents.

Although numerous mediators have been shown to increase neutrophil levels when injected into experimental animals (leukotriene B4, complement C5a), the initiation process (particularly monocytes–macrophages and endothelial cells) and triggering factor could, however, differ depending on the disease’s origin. Regardless of the disease type, these cells may be generated by the same unifying physiological mechanism that induces the inflammatory cascade. To identify and assess targeted interventions, there is a pressing need to better understand inflammatory signaling along with the cascade of specific mechanisms (12) and steps pertaining to the inflammatory process. Therefore, studying pro-inflammatory stimuli that elicit rapid transcriptional responses *via* transduced signals with the aim to master regulatory transcription factors proves determinant for apprehending the response sequences. TNF-α can induce a rapid global redistribution of chromatin activators to massive *de novo clustered* enhancer domains, with endothelial cells likely to play a major role in this process. Several endothelial dysfunction markers such as plasma endocan, a proteoglycan excreted by the endothelial cells, could be employed to monitor (13) the endothelial response to aggression. In the future, antioxidant enzymes, such as catalase and superoxide dysmutase, could represent (14) a strategy designed to protect organs and tissues from inflammation and oxidative stress. In addition, a recently published paper emphasized the role of monocyte subtypes Ly6C^low^, with these cells routinely patrolling the endothelial wall under steady-state conditions (15). These cells were shown to precede neutrophil arrival and orchestrate cell extravasation in response to TLR7/8-mediated vascular inflammation. The relative roles of monocytes and endothelial cells, along with their respective production of cytokines and chemokines, have yet to be clarified.

## Gender Differences in the Inflammatory Process

Sexual dimorphism is observed in inflammatory conditions all along the life course. While estrogens and androgens are sexual hormones known to modulate inflammation, their fluctuant levels in males and females of any age cannot account for the gender differences of the inflammation observed in humans and animals from birth to death. No uniform concept covering all inflammatory conditions could be found because of highly variable responses of the immune system to sexual hormones (16). Although estrogens are clearly known to modulate the immune response, in terms of cytokine production, receptors, and clinical outcome, these observations cannot fully explain the universal gender differences observed in acute inflammation, found across all age groups, from premature infants to geriatric patients.

In acute inflammatory conditions, male gender is associated with a higher risk of morbidity and mortality, with females at any age exhibiting better prognosis. However, in chronic inflammatory processes, less frequent than acute, females display worse prognosis and higher mortality, probably because of collateral tissue damages caused by higher inflammation (17). The longer the inflammation of a tissue lasts, the more damage is done. This could account for the higher mortality observed in females suffering from cystic fibrosis (18) and chronic obstructive pulmonary diseases (19). In chronic inflammation, we also reported worse prognosis in girls suffering from chronic asthma, cystic fibrosis, or sickle cell anemia (SCA) (20).

**Figure 1:**
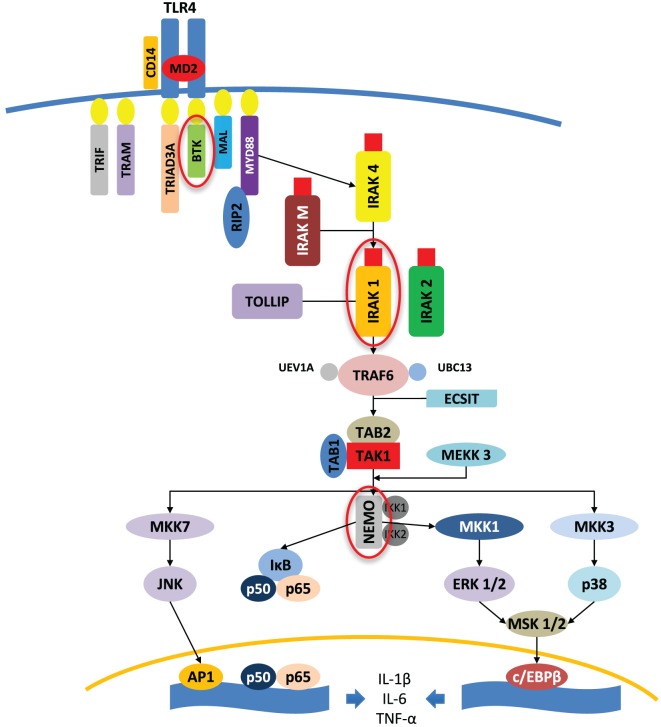
Protein kinases (circled in red) encoded by X-linked genes and involved in the TLR4 signaling pathway, adapted from the study by Akira et al. (22). An example of TLR4 signaling pathways is shown to highlight the implication of key X chromosome-linked kinases in the triggering of the inflammatory response. AP1, activator protein 1; BTK, Bruton’s tyrosine kinase; c/EBPβ, CCAAT/enhancer-binding protein β; ECSIT, evolutionary-conserved signaling intermediate in Toll pathway; ERK, extracellular signal-regulated MAP kinase; IKK, IκB kinase; IκB, inhibitor kappa B; IRAK, interleukin-1 receptor-associated kinase; JNK, c-Jun *N*-terminal kinase; MAL, myelin and lymphocyte; MAP, mitogen-activated protein; MD2, lymphocyte 96 antigen; MEKK, MAP/ERK kinase kinase; MKK, MAP kinase kinase; MSK, mitogen- and stress-activated kinase; MYD88, myeloid differentiation primary response 88; NEMO, NF-κB essential modulator; NF-κB, nuclear factor kappa B; p38, p38 MAP kinase; p50, NF-κB subunit 1; p65, NF-κB subunit 3 or RELA, v-rel avian reticuloendotheliosis viral oncogene homolog A; RIP2, receptor interacting protein-2; TAB, TAK1-binding protein; TAK, TGF-β-activated kinase; TIR, Toll/interleukin-1 receptor; TRAF, tumor necrosis factor receptor-associated factor; TRAM, translocating chain-associating membrane; TRIAD3/RNF216, ring finger protein 216; TRIF, TIR-domain-containing adapter-inducing interferon-β; TOLLIP, Toll interacting protein; UBC13, ubiquitin-conjugating enzyme 13; UEV1A, ubiquitin-conjugating enzyme variant 1A.

These observations have triggered gene analysis on the X chromosome, as well as investigating the potential influence of sexual steroids on inflammatory responses (21). In females, one of the X chromosomes is randomly silenced during X chromosome inactivation in the early stage of female embryogenesis, whereas the pseudoautosomal region of the X chromosome escapes inactivation. This process results in female cellular mosaicism, with half of the cells expressing genes derived from the maternal X chromosome and the other half expressing those derived from the paternal X chromosome. Moreover, spreading the inactivation signal on the pseudoautosomal region of the X chromosome may cause partial silencing of genes on the border, explaining higher gene expression of certain genes of the pseudoautosomal regions in males. The diversity in females is further increased because, if disadvantageous mutations occur in an X chromosome-linked gene, this will result in the functional loss of the respective protein in all cells of a male, but only in half of the cells in a female, resulting in differing regulatory responses and capacities. Finally, many mechanisms can hypothetically explain the better prognosis for females in acute inflammation (as infections), such as the expression of genes located on the non-recombining regions of the Y chromosome, sex hormone-mediated effects, differences in X-linked gene expressions of maternal or paternal origin, gene-dosage effects of sex chromosome-linked genes (namely, those genes that escape X chromosome inactivation or are reactivated), non-random X chromosome inactivation, and, finally, cellular mosaicism of females. The genes encoding some protein members of the TLR signaling pathway are linked to the X chromosome, such as IL-1 receptor-associated kinase 1, NF-κB essential modulator, and Bruton’s tyrosine kinase, with the Figure 1 adapted from the study by Akira et al.

Prepubescent children, displaying very low levels of sexual hormones, prove to represent a good model to evaluate the inflammatory response and clinical course of acute or chronic inflammatory diseases. We previously showed that in acute inflammation caused by pneumonia, pyelonephritis or bronchiolitis inflammatory markers (C-reactive protein or erythrocyte sedimentation rate) and neutrophil count were higher in females (23), suggesting better inflammatory recruitment in females during acute inflammation. This sexual dimorphism has been observed in all major disease categories, except for diseases of the musculoskeletal system and connective tissue in children younger than 20 years, with a higher mortality reported in males compared to females (24).

As similar observations were made by others in adults (25–27), this could explain the high number of studies reporting higher infections rates in males of any age, along with worse prognosis (28, 29). In septic shock, gender differences have commonly been reported, indicating that the males belong to most at-risk group (especially black in the United States) (30, 31). In other studies, women with sepsis exhibited lower age-specific incidence and mortality rates (32), being less frequently affected than males although with variable prognosis. This may perhaps be accounted for by underlying conditions, such as chronic respiratory failure, diabetes, or metastatic cancer, or by infection sites (33–35).

In children suffering from severe sepsis, we recently showed that girls tended to exhibit higher neutrophilic inflammation, longer fever duration, and lower pH on admission (36). In this study, the difference in neutrophil counts became significant on the third day after admission corresponding to the mean generation time of myelocytes. This observation points toward the origin of the difference being in the bone marrow rather than the marginated pool of neutrophils. In a previous work, we showed a different kinetic between males and females in terms of inflammatory cytokine production in whole blood stimulated with endotoxin, which could account for this gender difference becoming significant only on the third day from the beginning of the sepsis. The higher level of circulating neutrophils in girls could also contribute to the better pathogen clearance in girls during the early inflammatory response and consequently their better survival of sepsis. This is the first description of gender-related differences in the acid–base balance of children with sepsis. We have observed a significantly lower pH associated with a higher base deficit in girls at admission to the PICU. This difference could enhance the inflammatory response in females by increasing the expression of adhesion molecules and production of pro-inflammatory cytokines.

In adult patients with SCA, metabolic acidosis was likewise found with a much higher prevalence in women (52 versus 27% in men; *p* < 0.001) (35). Such acidosis, associated with several hemolytic markers and impaired ammonium availability, might contribute to the higher frequency of vasoocclusive crises and acute chest syndromes in girls with SCA (37).

## Genes Implicated in the Inflammatory Process

Inflammation is controlled by a highly coordinated gene expression program (37), involving numerous transcription factors, being potentially influenced by specific variables of the internal milieu like acid–base imbalance. This essential defense mechanism has developed early in metazoan evolution, as indicated by typical inflammatory responses to wounds in invertebrates like the starfish. This mechanism protects and organizes the symbiotic life of various cells in multicellular animals. While inflammation is a strong determinant for restoring the homeostatic balance in the body, it can, however, exceed its usual goals and cause major tissue damage such as in the event of shock sepsis. Such excessive inflammatory processes have accounted for the development of chronic inflammatory and autoimmune diseases. Therefore, an active knowledge and understanding of inflammatory processes appear essential, not only for improving the mechanisms’ efficiency in several acute diseases but also for preventing deleterious complications in a chronic setting.

In the presence of harmful agents that likely alter the organism’s integrity, a highly complex response is set in motion to restore the organism’s homeostasis (38). Genes involved in environmental and inflammatory responses have been shown to display an unusually high rate of duplication and loss during evolution (39). Recent technological advancements provide a clearer picture of the organizational principles underlying inflammatory gene expression. During the inflammatory response triggered upon stimulation (infection, burn, surgical procedure, etc.), several hundred genes are activated in a kinetically complex manner, either synchronously or only after many hours for some of them. Inducible recruitment of target genes, such as NF-κB, can apparently be influenced by a pre-existing chromatin state (40). The requirement for a chromatin-remodeling step at inflammatory genes has been shown to cause slower activation kinetics, while imposing the presence of additional transcription factors that induce the initial remodeling step. Many of these transcription factors involved in inflammatory processes can be selectively stimulated by a specific inflammation trigger, which lays the groundwork for stimulus specificity in genetic inflammation expression.

It seems now clear that genes activated by an identical trigger may differ extensively depending on cell types, even when the same cytokines are involved. The genomic regions that are active as enhancers in different cell types show only a slight overlap (41). Macrophages contain at least 35,000–45,000 identifiable genomic regions that are presently classified as enhancers. Regulatory mechanisms that control the inflammatory process designed to lessen potential tissue damage must have been positively selected in the course of evolution. A central role is played by the B-cell lymphoma 6 protein (BCL6), a sequence-specific transcriptional repressor known for its role in both B-cell differentiation and B-cell lymphomas. The BCL6 has been shown to prevent excessive production of a large fraction of lipopolysaccharide-inducible genes (42). Currently, the entire set of players involved in the inflammatory response is still incompletely defined and thus largely unknown.

In immune challenges, especially if acute, females exhibit a better prognosis and survival than males at any age. In inflammatory processes, the immune response relies on the heterogeneity of immune cells, along with their ability to respond to pathogen challenges (43), with lymphocytes displaying a highly diverse antigen receptor repertoire that matches pathogen diversity. In addition, the inflammatory response is under the influence of epigenetic regulation, which requires flexible adaptation to diverse environmental challenges like pH variations.

## Concluding Remarks

There is evidence favoring the existence of links between acid–base balance and cytokine concentrations, with acidosis as potential unifying factor for the trigger threshold of the inflammatory response. Gender differences in the inflammatory response could be linked to the acid–base balance of the cellular environment that influences the expression of genes related in particular to the X chromosome. Endothelial cells may play a fundamental role in this process by sensing acid–base fluctuations. Further understanding of their potent role in the initiation of the inflammatory cascade could help design new strategies to interfere with the inflammatory process.

## Author Contributions

The review results from the discussion and the consensus of all authors listed (GC, NL, FC, JD, and MC). Literature review on the topic was analyzed and produced by GC, NL, and MC. The review was written by GC.

## Conflict of Interest Statement

The authors declare that the research was conducted in the absence of any commercial or financial relationships that could be construed as a potential conflict of interest.

## Abbreviations

TNF-α, tumor necrosis factor-α; IL-1β, interleukin-1β; IL-6, interleukin-6; GLUT 2, glucose transporter 2; NF-κB, nuclear factor kappa B; BCL6, B-cell lymphoma 6 protein; G-CSF, granulocyte colony-stimulating factor.
